# Allergic rhinitis and rhinosinusitis synergistically compromise the mental health and health-related quality of life of Korean adults: A nationwide population-based survey

**DOI:** 10.1371/journal.pone.0191115

**Published:** 2018-01-11

**Authors:** Ji-Hyeon Shin, Daeyoung Roh, Dong-Hee Lee, Soo Whan Kim, Sung Won Kim, Jin Hee Cho, Byung-Guk Kim, Boo-Young Kim

**Affiliations:** 1 Department of Otolaryngology-Head and Neck Surgery, College of Medicine, The Catholic University of Korea, Seoul, Republic of Korea; 2 Department of Psychiatry, Chuncheon Sacred Heart Hospital, Hallym University College of Medicine, Chuncheon-si, Gangwon-do, Republic of Korea; 3 Mind-neuromodulation Laboratory, Hallym University College of Medicine, Chuncheon-si, Gangwon-do, Republic of Korea; Ospedale S. Corona, ITALY

## Abstract

**Background:**

Allergic rhinitis (AR) and rhinosinusitis (RS) negatively impact psychological well-being and health-related quality of life (HRQoL). However, few population-based studies have investigated the effects of these conditions on mental health and HRQoL.

**Purpose:**

To explore independent associations of AR and/or RS with mental health and HRQoL using data from the 2013–2015 Korea National Health and Nutrition Examination Survey (KNHANES).

**Methods:**

The KNHANES is a nationwide cross-sectional survey of the non-institutionalized population of Korea. A total of 15,441 adults completed the clinical examination and the health questionnaire. We divided all participants into four groups: AR-/RS-, AR-/RS+, AR+/RS-, and AR+/RS+. Logistic regression analyses were performed after adjustment for sociodemographic characteristics, general health behaviors, and other comorbidities.

**Results:**

The AR+/RS+ group contained the highest proportion of subjects with perceived stress and depressed mood. Subjects with AR+/RS+ also had more frequent problems in terms of pain/discomfort and anxiety/depression. After adjusting for all confounders, the odds ratios (ORs) were 2.96 (p = 0.009) for depressed mood and 3.17 (p = 0.013) for suicidal ideation in the AR+/RS+ group compared with in the AR-/RS- group. The AR+/RS- group reported more perceived stress (OR, 1.56, p = 0.003) and depression (OR, 1.72, p = 0.024) compared with the AR-/RS- group. In terms of the ORs for HRQoL, the AR+/RS+ group reported more problems in terms of self-care (OR, 3.73, p = 0.038) and more pain/discomfort (OR 2.19, p = 0.006) compared with the AR-/RS- group.

**Conclusions:**

In the Korean population, AR and RS exerted a synergistic negative impact on mental health and HRQoL, especially suicidal ideation. Most patients seek help from clinicians for impaired HRQoL. Therefore, clinicians should consider the underlying mental health and HRQoL of patients with AR and/or RS, as these may be impaired by their conditions.

## Introduction

Clinicians evaluate the health problems of patients from the viewpoint of anatomical/functional impairment or disability/handicap. The latter aspect of health-related problems is difficult to qualify but is directly related to health and life. Therefore, the World Health Organization Quality of Life (WHOQOL) Group has defined quality of life (QoL) as an individual’s perception of his/her position in life in the context of the culture and value systems in which that individual lives and as it relates to that individual’s goals, expectations, standards, and concerns [1]. This concept covers broadly all aspect of an ill patient’s life as human. Health-related quality of life (HRQoL) refers to the physical, psychological, and social domains of health considered as distinct areas influenced by individual experiences, beliefs, expectations, and perceptions [2–4]. The HRQoL has become an important index in terms of medical treatment and clinical care. The HRQoL integrates both objective functioning and subjective evaluations of the health of an individual. It is important that a patient’s experience with both disease and treatment is directly assessed to allow for a comprehensive understanding of health status [5]. Clinicians must treat the clinical problems afflicting their patients and also manage other problems for which patients are seeking help, as reflected in their QoL. Thus, both clinicians and researchers must focus on HRQoL when deciding on appropriate treatments or interventions for individual patients.

Allergic rhinitis (AR) is an IgE-mediated inflammatory disease of the nasal mucosa. AR is one of the most common and burdensome medical conditions in many countries. AR affects 10–40% of the population worldwide, and its prevalence has recently rapidly increased. The socioeconomic burden imposed by AR is significant, as AR increases both direct and indirect costs, burdening hospitals and compromising work and academic performance [6–9].

Rhinosinusitis (RS) is an inflammation of the nose and the paranasal sinuses. RS is categorized according to disease duration as acute rhinosinusitis (ARS) or chronic rhinosinusitis (CRS) [10]. CRS is one of the most common chronic medical conditions, affecting approximately 10–15% of subjects worldwide. As is true of AR, RS also imposes a large socioeconomic burden and increases both the direct costs of medical and surgical treatment and the indirect costs caused by decreased productivity [11–13]. Patients with AR and/or CRS suffer not only from nasal symptoms but also extra-nasal symptoms, including fatigue, cognitive dysfunction, sleep disturbance, mood disturbance, and poor work productivity. AR and CRS affect physical, functional, and psychological activities, compromising mental health and QoL [6, 7, 14–18].

Many clinicians report that they frequently encounter AR and RS in the same patients but cannot tell which condition is more problematic. Although a mutual relationship may exist between AR and RS, little evidence supports this hypothesis. Additionally, the co-existence of AR and RS is associated with more severe symptoms, compromising psychological health and QoL [19–21]. However, few studies have explored mental health and HRQoL in terms of the presence or absence of both AR and RS in the general population. We explored the association(s) of AR and/or CRS with mental health and HRQoL in subjects with AR, RS, or both and compared these data with those from controls using data from a nationwide population-based study.

## Materials and methods

### Study population

The KNHANES is a nationwide, population-based, cross-sectional health examination and survey that has been conducted by the Division of Chronic Disease Surveillance under the auspices of the Korea Centers for Disease Control and Prevention (KCDC) since 1998. The KNHANES was designed to assess the health and nutritional status of Koreans, to monitor trends in health risk factors and the prevalence of major chronic diseases, and to provide data allowing the development and evaluation of health policies and programs in Korea. The target population is non-institutionalized Korean citizens residing in Korea. The survey consists of three components; a health interview, a nutritional survey, and a health examination survey. The health interview and health examination are performed by trained medical staff and interviewers in mobile examination centers. The nutritional survey is conducted in the homes of subjects 1 week after the health interview.

We used data collected during the 2013–2015 KNHANES. Of the 18,034 adults (> 19 years of age) who participated in the survey, 15,441 completed both the clinical examination and the health questionnaire. All subjects provided written informed consent for use of their data prior to survey commencement. The study was approved by the Institutional Review Board of the Korea Centers for Disease Control and Prevention.

### Definitions of AR and RS

All subjects were asked about their AR and RS histories. AR was addressed with the following question: “Have you ever been diagnosed with allergic rhinitis by a doctor?” Subjects who answered “yes” were considered to have AR. RS was addressed with a similar question: “Have you ever been diagnosed with rhinosinusitis by a doctor?” Subjects who answered “yes” were considered to have RS. We divided all subjects into the following four groups: AR-/RS-, AR-/RS+, AR+/RS-, and AR+/RS+.

### Mental health survey

The mental health survey included three dimensions: perceived stress, depressed mood, and suicidal ideation. Each subject reported the level of stress experienced as none, mild, moderate, or severe. Subjects with moderate-to-severe stress were considered to exhibit stress. Each subject answered the following question to assess depressed mood: “Have you ever felt sad or despair for 2 consecutive weeks during the past year such that your daily life was hindered?” Subjects who answered “yes” were considered to exhibit a depressed mood. To assess suicidal ideation, each subject was asked the following question: “Have you ever thought about committing suicide in the last year?” Subjects who answered “yes” were considered to exhibit suicidal ideation.

### HRQoL survey

The EuroQoL 5-dimension (EQ-5D) is a brief, self-report, standardized, non-disease-specific instrument used to measure HRQoL along the following five dimensions: mobility, self-care, ability to perform usual activities, pain/discomfort, and anxiety/depression. Each dimension has three levels: no problems, some problems, and extreme problems. All subjects were asked to indicate their health status by checking the box next to the most appropriate statement in each of the five dimensions [22, 23]. Subjects who indicated some or extreme problems in a dimension were considered to experience poor QoL in that dimension.

### Sociodemographic and clinical characteristics

During the health interviews, data were collected using self-report questionnaires. The health interview evaluated educational level, occupation, household income, marital status, residential area, smoking status, alcohol consumption, exercise status, previous and present diseases, activity limitations, sleep duration, and subjective health. Smoking history was categorized as current smoker, ex-smoker, or nonsmoker. Alcohol use was based on the amount of alcohol consumed per day during the month before the interview. Male subjects who drank seven or more glasses of alcohol more than twice per week and female subjects who drank five or more glasses more than twice per week were categorized as heavy drinkers. Regular exercise was defined as moderate physical activity performed for at least 30 minutes at a time at least five times a week. Height, weight, and waist circumference were measured by well-trained medical professionals. The body mass index (BMI) was the body weight (kg) divided by the height squared (m^2^). Obesity was defined as a BMI ≥ 25 kg/ m^2^.

### Statistical analysis

Statistical analyses were performed using the complex sample analysis program of PASW 18.0 (SPSS Inc., Chicago, Illinois, USA) to reflect the complex sampling design and sampling weights of the KNHANES and to provide nationally representative prevalence estimates. Results are presented as percentages ± standard errors for categorical variables and as estimated means ± standard errors for continuous variables. To compare categorical and continuous variables, the chi-square test and a general linear model, respectively, were used. The multivariate analysis employed logistic regression to explore associations of AR and RS with mental health and EQ-5D score. We also calculated adjusted odds ratios (ORs) for mental health and the EQ-5D score. We adjusted for age and gender (model 1) and then for these variables plus educational level, marital status, household income, occupation, residential area, smoking status, alcohol consumption, exercise status, sleep duration, and obesity (model 2). Next, we adjusted for all covariates in model 2 plus comorbidities (hypertension, diabetes, stroke, ischemic heart disease, arthritis, asthma, atopic dermatitis, thyroid disease, liver cirrhosis, chronic renal failure, and depression), activity limitations, subjective health, and total cholesterol and triglyceride levels (model 3).

## Results

### Sociodemographic and clinical characteristics

Of the 15,441 subjects, 2,286 were diagnosed with AR and 1,189 with RS, yielding weighted prevalences of 14.7% and 7.8%, respectively. The weighted prevalences of AR-/RS-, AR-/RS+, AR+/RS-, and AR+/RS+ status were 80.6%, 4.6%, 11.7%, and 3.1%, respectively.

**Fig 1:**
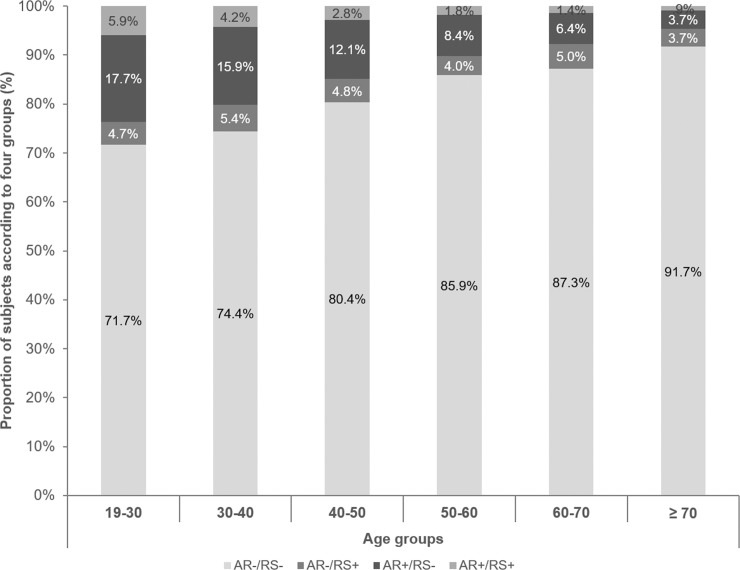
Distribution of the four groups by age. The proportions of AR+/RS- and AR+/RS+ patients decreased as a function of age.

Based on age at examination, all subjects were divided into the following six age groups: 19–29, 30–39, 40–49, 50–59, 60–69, and 70 years and older. The proportions of subjects in the AR+/RS- and AR+/RS+ groups tended to decrease as a function of age (Fig 1). The average ages were as follows: 47.4 ± 0.3 years in the AR-/RS- group, 44.9 ± 0.7 years in the AR-/RS+ group, 39.3 ± 0.4 years in the AR+/RS- group, and 37.4 ± 0.7 years in the AR+/RS+ group. The AR-/RS- subjects were oldest, and the AR+/RS+ subjects youngest. Overall, subjects without AR were older than those with RS.

**Fig 2:**
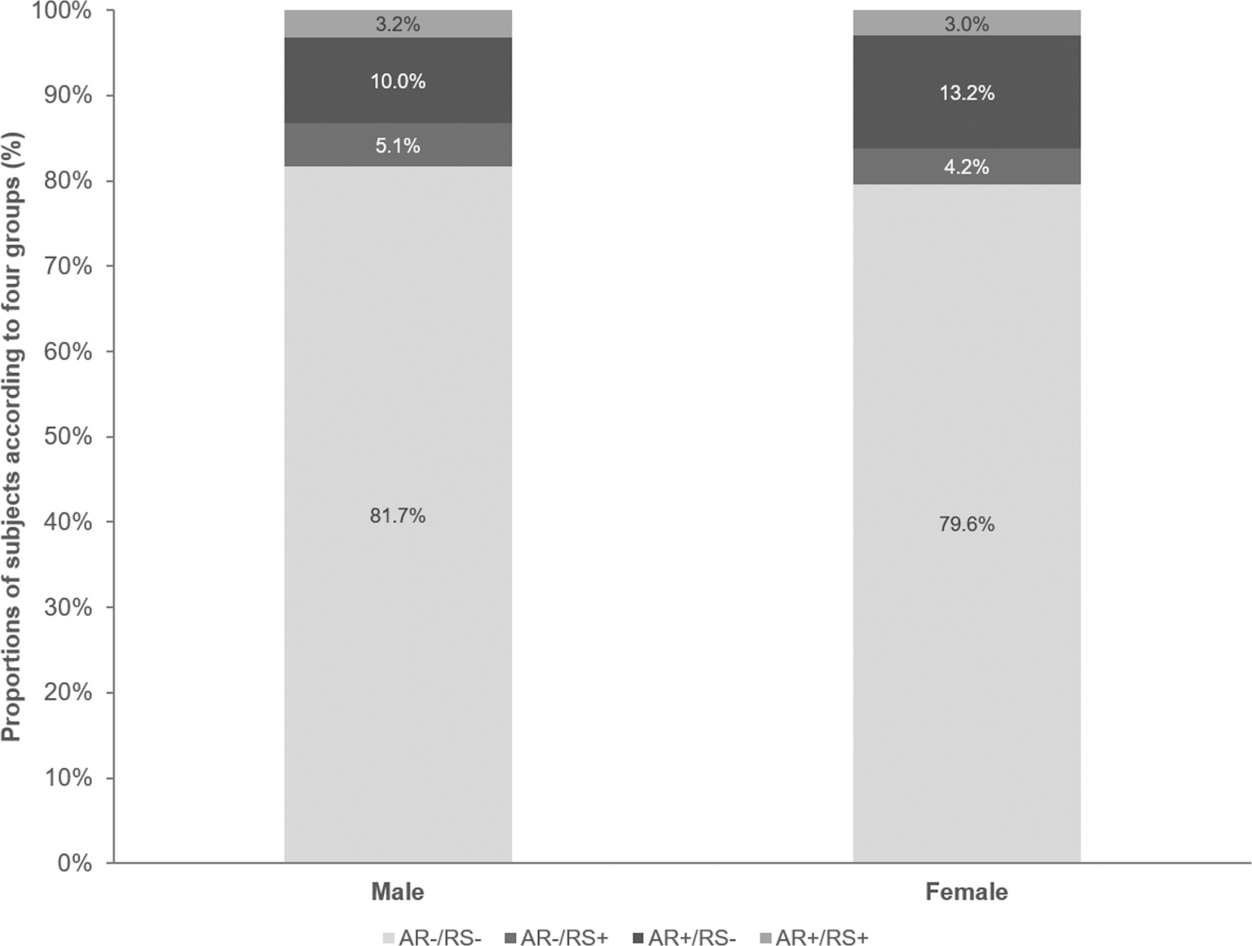
Distribution of the four groups by gender. More AR+/RS- patients were female than male.

Males constituted a higher proportion of the AR-/RS- group (81.7% vs. 79.6%) and a lower proportion of the AR+/RS- group (10.0% vs. 13.2%) than females (Fig 2).

The sociodemographic and clinical characteristics by group are shown in Table 1. Subjects without AR (with or without RS) were more likely to be male; to have a low educational level and household income; to have no spouse; to be obese; to report activity limitations; to exhibit high total cholesterol and triglyceride levels; and to have a high prevalence of hypertension, diabetes, stroke, and arthritis compared to those with AR. Subjects with RS (with or without AR) were more likely to report urban residence, short sleep duration, depression, and bad subjective health and discomfort compared to those without RS. The prevalences of asthma and atopic dermatitis were highest in the AR+/RS+ group, followed by the AR+/RS-, AR-/RS+, and AR-/RS groups. Additionally, subjects with AR were more likely to have asthma and atopic dermatitis but less likely to have hypertension and diabetes compared to those with RS.

**Table 1:** Sociodemographic and clinical characteristics by presence of allergic rhinitis and/or rhinosinusitis.

	AR-/RS- (N = 12,445)	AR-/RS+ (N = 710)	AR+/RS- (N = 1,807)	AR+/RS+ (N = 479)	p value
Age (years)	47.4 ± 0.3	44.9 ± 0.7	39.3 ± 0.4	37.4 ± 0.7	< .0001*
Gender (male, %)	49.4 ± 0.4	54.4 ± 2.3	42.3 ± 1.4	46.8 ± 2.9	< .0001*
Educational level (≤ middle school, %)	28.3 ± 0.7	21.6 ± 1.8	14.0 ± 0.9	12.8 ± 1.8	< .0001*
Without spouse (%)	14.6 ± 0.5	13.2 ± 1.6	9.7 ± 1.0	8.2 ± 1.7	< .0001*
Income (lower quartile, %)	16.4 ± 0.6	14.8 ±1.7	9.0 ± 0.9	11.0 ± 1.9	< .0001*
Without occupation (%)	37.5 ± 0.6	35.8 ± 2.1	38.3 ± 1.3	35.5 ± 2.8	0.668
Residential area (urban, %)	70.4 ± 1.1	73.8 ± 2.4	73.4 ± 1.8	75.3 ± 2.9	0.047*
Current smoking (%)	47.4 ± 0.9	38.2 ± 3.1	42.7 ± 2.7	36.8 ± 5.1	0.008*
Heavy drinking (%)	14.1 ± 0.4	14.3 ± 1.6	11.5 ± 1.0	13.3 ± 2.4	0.181
Regular exercise (%)	17.3 ± 0.4	18.9 ± 1.8	18.7 ± 1.2	17.1 ± 2.1	0.562
Short sleep duration (≤ 6 hours)	43.1 ± 0.5	46.5 ± 2.2	39.8 ± 1.4	47.3 ± 2.8	0.018*
Obesity (BMI ≥ 25 kg/m^2^, %)	33.1 ± 0.5	32.2 ± 2.0	26.7 ± 1.3	28.3 ± 2.6	< .0001*
Hypertension (%)	18.9 ± 0.4	16.4 ± 1.6	10.4 ± 0.8	6.9 ± 0.5	< .0001*
Diabetes (%)	7.6 ± 0.3	6.5 ±0.9	3.4 ± 0.4	2.9 ± 0.9	< .0001*
Stroke (%)	2.1 ± 0.1	2.1 ± 0.6	0.5 ± 0.1	1.0 ± 0.4	< .0001*
Ischemic heart disease (%)	3.5 ± 0.7	1.2 ± 0.2	0.5 ± 0.2	1.9 ± 0.1	0.001*
Arthritis (%)	10.3 ± 0.3	11.7 ± 1.3	7.6 ± 0.7	9.1 ± 1.5	0.006*
Asthma (%)	2.2 ± 0.2	4.2 ± 0.9	6.3 ± 0.7	11.1 ± 1.8	0.001*
Atopic dermatitis (%)	2.4 ± 0.2	2.4 ± 0.6	6.9 ± 0.8	9.4 ± 1.6	0.001*
Liver cirrhosis (%)	0.3 ± 0.1	0.2 ± 0.1	0.2 ± 0.1	0.3 ± 0.2	0.753
Chronic renal disease (%)	0.4 ± 0.1	0.3 ± 0.1	0.5 ± 0.2	0.5 ± 0.3	0.882
Depression (%)	4.0 ± 0.2	5.6 ± 1.0	5.2 ± 0.6	7.9 ± 1.7	0.001*
Activity limitations (%)	7.5 ± 0.3	10.8 ± 1.3	5.6 ± 0.6	6.7 ± 1.3	0.001*
Subjective health (%)	16.9 ± 0.4	21.2 ± 1.9	16.5 ± 1.1	20.7 ± 2.2	0.025*
Total cholesterol (≥240 mg/dL, %)	8.3 ± 0.3	9.5 ± 1.4	5.9 ± 0.7	6.7 ± 1.5	0.028*
Triglyceride (≥200 md/dL, %)	17.0 ± 0.4	17.5 ±1.8	12.0 ± 1.0	11.0 ± 2.2	< .0001*

BMI: body mass index

*: p < 0.05

### Mental health and EQ-5D scores by AR and/or RS status

**Fig 3:**
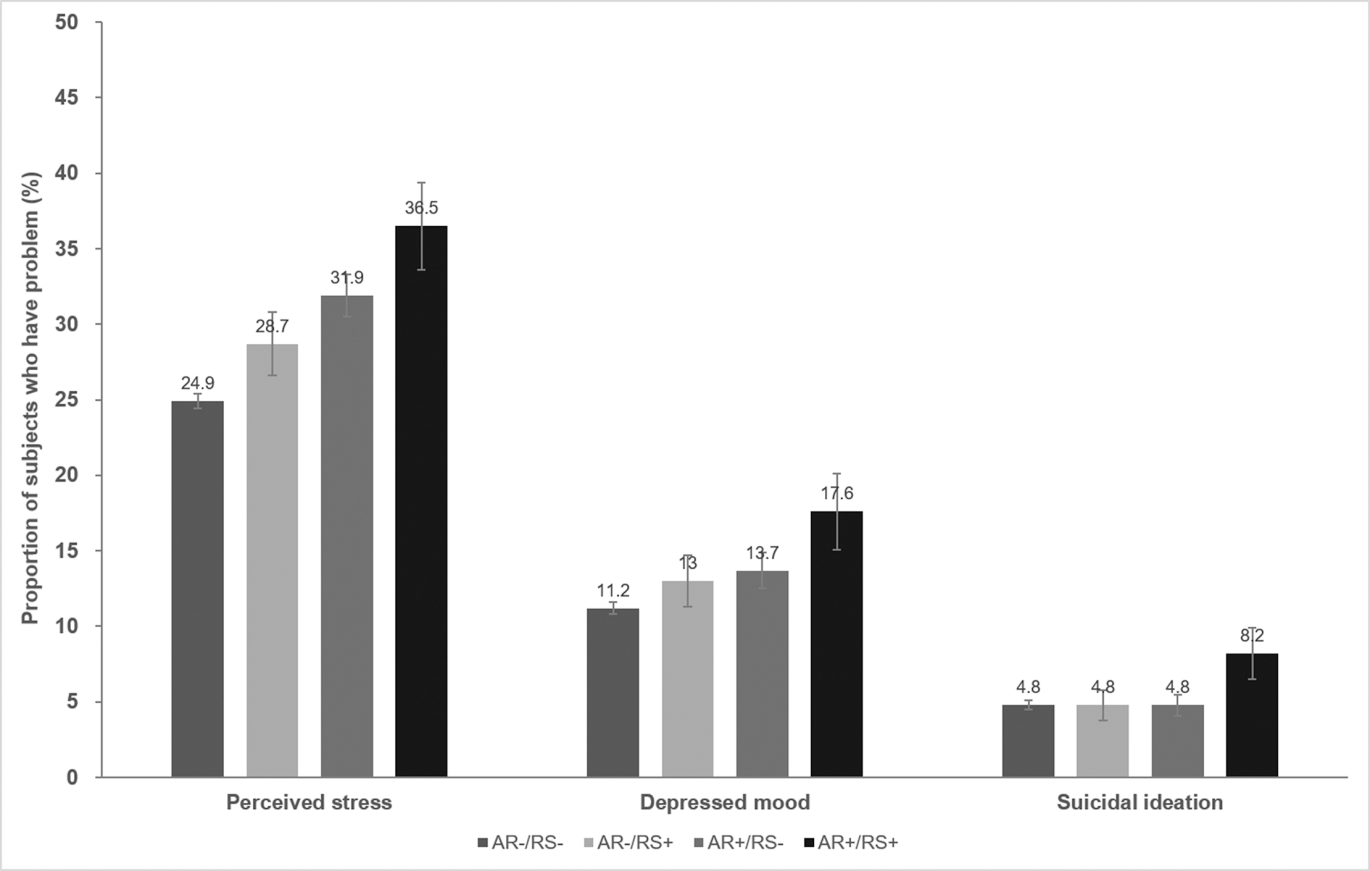
Proportions of subjects reporting problems with their mental health by the presence and/or absence of allergic rhinitis and rhinosinusitis.

Fig 3 shows the mental health characteristics of the four groups. The AR+/RS+ group had the highest proportions of subjects with perceived stress (36.5%, p < 0.0001) and depressed mood (17.6%, p = 0.004), followed by the AR+/RS-, AR-/RS+, and AR-/RS- groups, in that order. However, the extent of suicidal ideation did not differ significantly among groups. The AR-only group had higher proportions of participants with perceived stress and depressed mood compared with the RS-only group.

**Fig 4:**
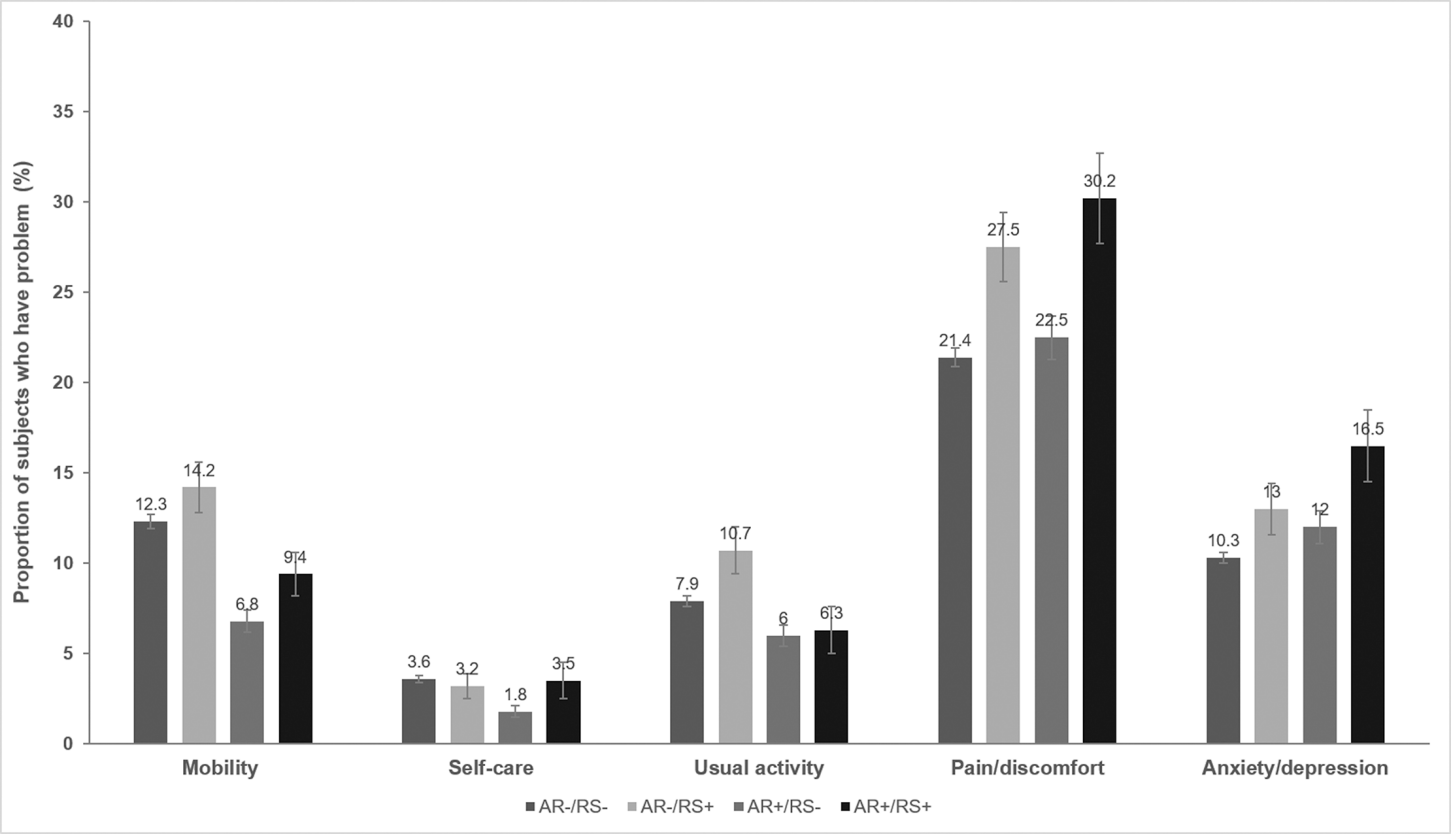
Proportions of subjects reporting problems in their health-related quality of life by the presence and/or absence of allergic rhinitis and rhinosinusitis.

In terms of the HRQoL, Fig 4 shows the EQ-5D scores of the four groups. The proportions of subjects reporting problems in terms of mobility (14.2%, p < 0.0001) and the performance of usual activities (10.7%, p = 0.002) were highest in the AR-/RS+ group, followed by the AR-/RS-, AR+/RS+, and AR+/RS- groups. The AR-/RS- group had the highest proportion of subjects reporting problems in terms of self-care (3.6%, p = 0.002), followed by the AR+/RS+, AR-/RS+, and AR+/RS- groups. The AR+/RS+ group had the highest proportion of participants with pain/discomfort and anxiety/depression (30.2% and 16.5%, respectively, both p < 0.000), followed by the AR-/RS+, AR+/RS-, and AR-/RS- groups. Subjects with only RS had more problems in all dimensions of the EQ-5D than did those with only AR.

### Associations of mental health and HRQoL with AR and/or RS

**Fig 5:**
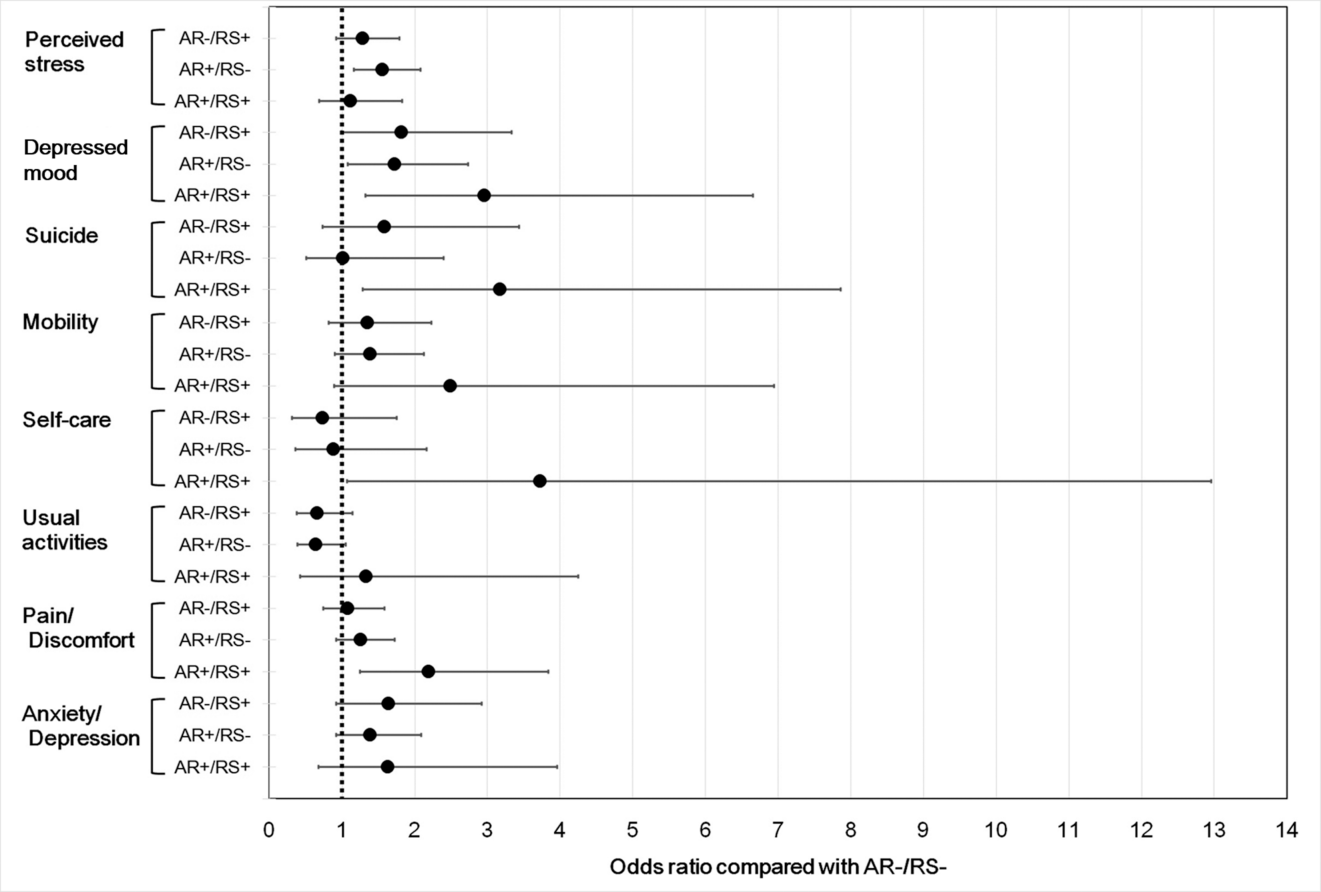
Associations of allergic rhinitis and rhinosinusitis with mental health and health-related quality of life according to adjusted odds ratios: comparison with the AR-/RS- group.

Table 2 shows the associations of mental health and HRQoL with AR and/or RS according to multiple logistic regression analysis. After adjustment for all confounding factors, including sociodemographic and clinical characteristics, health-related behaviors, and the presence of comorbidities (model 3), the ORs for AR+/RS+ group were 2.96 (95% CI, 1.32–6.65, p = 0.009) for depressed mood and 3.17 (95% CI, 1.28–7.86, p = 0.013) for suicidal ideation compared with the AR-/RS- group. In the AR+/RS- group, the ORs were 1.56 (95% CI, 1.162–2.082, p = 0.003) for perceived stress and 1.72 (95% CI, 1.08–2.73, p = 0.024) for depressed mood compared with the AR-/RS- group. In terms of the HRQoL, only the AR+/RS+ group exhibited a significantly increased OR on the EQ-5D. The AR+/RS+ group reported more problems in terms of self-care (OR, 3.73; 95% CI, 1.07–12.96, p = 0.038) and pain/discomfort (OR, 2.19; 95% CI, 1.25–3.84, p = 0.006) compared with the AR-/RS- group (Fig 5).

**Table 2:** Associations of allergic rhinitis and rhinosinusitis with mental health and health-related quality of life according to adjusted odds ratios (with confidence intervals).

	AR-/RS-	AR-/RS+	AR+/RS-	AR+/RS+
**Model 1**				
Perceived stress	1	1.18 (0.96–1.46)	1.24 (1.08–1.42) *	1.50 (1.17–1.93) *
Depressed mood	1	1.25 (0.91–1.70)	1.32 (1.06–1.64) *	1.86 (1.30–2.66) *
Suicidal ideation	1	1.05 (0.67–1.65)	1.13 (0.81–1.56)	2.10 (1.34–3.29) *
Mobility	1	1.55 (1.16–2.04) *	0.95 (0.76–1.19)	1.76 (1.17–2.65) *
Self-care	1	1.09 (0.68–1.74)	0.96 (0.65–1.42)	2.45 (1.36–4.44) *
Usual activities	1	1.75 (1.29–2.37) *	1.25 (0.97–1.61)	1.57 (1.01–2.43) *
Pain/Discomfort	1	1.57 (1.28–1.94) *	1.33 (1.14–1.54) *	2.21 (1.73–2.82) *
Anxiety/Depression	1	1.10 (1.10–1.88) *	1.38 (1.16–1.65) *	2.16 (1.61–2.91) *
**Model 2**				
Perceived stress	1	1.30 (0.94–1.81)	1.52 (1.15–2.01) *	1.13 (0.70–1.82)
Depressed mood	1	1.77 (0.99–3.18)	1.67 (1.08–2.57) *	2.79 (1.32–5.93) *
Suicidal ideation	1	1.45 (0.65–3.23)	1.10 (0.55–2.18)	3.11 (1.27–7.63) *
Mobility	1	1.42 (0.89–2.26)	1.24(0.83–1.85)	2.51 (1.08–5.84) *
Self-care	1	0.96 (0.35–2.63)	1.00 (0.49–2.06)	4.03 (1.49–10.86) *
Usual activities	1	1.80 (1.11–2.95) *	1.47 (0.93–2.33)	1.26 (0.62–2.57)
Pain/Discomfort	1	1.24 (0.87–1.77)	1.24(0.92–1.68)	2.35 (1.35–40.83) *
Anxiety/Depression	1	1.68 (0.99–2.84)	1.35 (0.92–1.97)	1.73 (0.81–3.70)
**Model 3**				
Perceived stress	1	1.28 (0.92–1.79)	1.56 (1.16–2.08) *	1.12 (0.69–1.83)
Depressed mood	1	1.82 (0.99–3.33)	1.72 (1.08–2.73) *	2.96 (1.32–6.65) *
Suicidal ideation	1	1.58 (0.73–3.44)	1.01 (0.51–2.40)	3.17 (1.28–7.86) *
Mobility	1	1.35 (0.82–2.23)	1.39 (0.90–2.13)	2.49 (0.89–6.94)
Self-care	1	0.73 (0.31–1.75)	0.88 (0.36–2.16)	3.73 (1.07–12.96) *
Usual activities	1	0.66 (0.38–1.14)	0.64 (0.39–1.05)	1.33 (0.42–4.25)
Pain/Discomfort	1	1.08 (0.74–1.58)	1.26 (0.92–1.72)	2.19 (1.25–3.84) *
Anxiety/Depression	1	1.64 (0.92–2.92)	1.39 (0.92–2.09)	1.63 (0.68–3.96)

Model 1: Adjusted for age and gender.

Model 2: Adjusted for the variables in model 1 plus educational level, marital status, household income, occupation, residential area, smoking status, alcohol consumption, exercise status, sleep duration, and obesity.

Model 3: Adjusted for the variables in model 2 plus the comorbidities of hypertension, diabetes, stroke, ischemic heart disease, arthritis, asthma, atopic dermatitis, thyroid disease, liver cirrhosis, chronic renal failure, and depression; activity limitations; subjective health; and total cholesterol and triglyceride levels.

*: p < 0.05

## Discussion

This population-based nationwide study examined the impact of AR and/or RS in terms of mental health and HRQoL. Several studies have investigated mental health or QoL in patients with AR or RS. However, as many of these were cohort studies, they may have been affected selection bias; also, they did not include non-referred subjects. Additionally, although AR and RS comorbidity is common [24, 25], few studies considered the possibly synergistic effect of the condition on mental health and QoL [14, 20, 26–28]. Our present study is, to the best of our knowledge, the first population-based study to investigate mental health and the HRQoL of Koreans with AR and/or RS in consideration of a possible interaction between AR and RS.

In the present study, the prevalence of AR in Korean subjects was 14.7%, and it decreased with age during 2013–2015. The prevalence was thus slightly less than the 16.2% of the 2010 KNHANES [29]. In another study using 2008–2012 KNHANES data, the prevalence of AR diagnosed on the basis of allergy testing was similar (14.9%) to our figure, whereas the prevalence of symptom-based AR was higher (26.6%) than we found. The 2008–2012 KNHANES also showed that the prevalence of had decreasing pattern by aging [30]. The prevalence of RS was 7.8% in our present study, which is similar to those in other studies of the general Korean population (range = 6.95–10.78%) [31, 32].

We found that 40.2% of subjects with RS also had AR. In two Korean population-based studies, the proportions of patients with comorbid AR and RS were 33.6% [32] and 18.4% [29], respectively. Several studies have found that atopy was more prevalent in CRS patients, and positive skin test results were reported in 50–84% of such patients [24]. A recent population-based study performed in the United States reported that the prevalence of comorbid AR in CRS patients was 35.1% [33].

In the present study, we found that AR and RS negatively affected mental health. The AR+/RS+ group contained the highest proportions of subjects with perceived stress (36.5%, p < 0.0001) and depressed mood (17.6%, p = 0.004). The AR-only group contained slightly higher proportions of subjects with perceived stress and depressed mood than did the RS-only group. However, the extent of suicidal ideation did not differ among the groups.

We also used multivariate logistic regression analysis to examine the association(s) of AR and/or RS with mental health. After adjusting for all confounders, we found that the AR+/RS- group exhibited more positive associations with perceived stress and depressed mood compared with the AR-/RS- group. Thus, AR had a more negative effect on perceived stress and depressed mood than RS. More surprisingly, the AR+/RS+ group exhibited about three-fold the proportions of those with depressed mood and suicidal ideation than did the AR-/RS- group (ORs = 2.96 and 3.17, respectively). Thus, AR and RS seemed to exert a negative synergistic effect on mental health. Additionally, our finding that comorbid AR and RS increased the risk of suicidal ideation has major implications for Korea, because South Korea’s suicide rate is the highest among member countries of the Organization for Economic Co-operation and Development (OECD) [34].

Previous studies found that AR and RS compromised mental health. In addition to nasal symptoms, AR patients experience fatigue, headache, mood and sleep disturbances, physical and mental problems, and cognitive dysfunction [35, 36]. A recent study in Korea found that AR patients were at higher risk of stress and depressed mood and required more psychological consultations; persistent and severe AR was associated with poor mental health [37]. A large population-based study performed in the United States showed that AR and CRS increased the risks of limitations in activities, work, and social interactions and that AR was also associated with cognitive limitations [20]. In the general Korean population, it was reported that high-level stress increased the prevalence of both AR and CRS [31, 38].

In the present study, we found that AR and RS both exerted negative impacts on HRQoL. Subjects without AR, with or without RS, reported more problems in terms of mobility and the performance of usual activities than did those with AR. However, subjects in the AR-/RS- group had a higher proportion of self-care problems than did those in the AR+/RS- group, which may have been attributable to differences in age distribution. As the AR-/RS- group had the oldest average age, such subjects may have experienced more difficulties in terms of self-care than those in other groups. Also, the AR-/RS+ group (the next-oldest group) reported problems with mobility and the performance of usual activities. In the pain/discomfort and anxiety/depression dimensions, the AR+/RS+ group had the highest proportions of participants reporting problems. The groups with RS, with or without AR, contained higher proportions of subjects with problems in the pain/discomfort and anxiety/depression dimensions compared to AR subjects with or without RS. According to the logistic regression analysis, the AR+/RS+ group faced increased risks of poor self-care and pain/discomfort compared with the AR-/RS- group, with ORs of 3.73 and 2.19, respectively, after adjusting for all confounders. These results also emphasized the synergistic effect of AR and RS on self-care problems and pain/discomfort, as was also true in terms of depressed mood and suicidal ideation.

Previous studies also found that AR or RS impaired HRQoL. A systematic review found that the symptoms of AR negatively affected HRQoL [36]. Crump et al. concluded that the severity of CRS symptoms was negatively associated with EQ-5D score in Canadian adults [39]. A cross-sectional study performed in Nigeria showed that the severity of CRS negatively impacted HRQoL, particularly the physical domain [40]. In a Chinese population-based study, subjects with self-reported CRS exhibited impaired HRQoL in both the physical and mental domains [41]. In a population-based study in Korea, Kim et al. showed that CRS was positively associated with a poor HRQoL after controlling for confounders [42].

The mechanisms by which AR and CRS negatively affect mental health and HRQoL are not clear. Previous studies found that some nasal and extra-nasal symptoms had negative impacts on mental health and HRQoL. Olfactory loss, a severe symptom of AR or CRS, was associated with anxiety, depressed mood, and suicidal ideation [26, 43]. The symptoms of AR have adverse effects on sleep, daily activities, physical and mental status, and social functioning and reduce HRQoL [36, 44]. Some studies on CRS found that the associated pain, depressed mood, and sleep disturbance reduced HRQoL [28, 41, 45]. Thus, these results suggest that olfactory dysfunction and extra-nasal symptoms including pain, sleep disturbance, and depressed mood, may contribute to a deterioration in mental health and HRQoL as indicated in our results.

Meanwhile, we assumed the mechanisms of deterioration effect of AR and RS on mental health and HRQoL, in terms of immunologic reaction. Several studies have investigated the associations of the cytokines involved in allergic and RS-related inflammation with mental health and HRQoL. Well-known inflammatory cytokines characteristic of AR or RS, such as IL-6 and IFN-γ [46, 47], have been shown to be linked to depression via modulation of central nervous system (CNS) processes. Peripheral cytokines, such as IL-6, potentially cross the blood–brain barrier, thus affecting the brain. The nasal cavity is directly connected to the CNS via the olfactory nerve [48, 49]. A recent study found that IL-6 was elevated in patients with seasonal AR, and this was related to depressed mood and poor sleep quality [50]. A review reported that elevated IL-6 was associated with suicidal ideation and suicide [51]. IFN-γ and many other cytokines have been found to reduce the availability of serotonin, low levels of which are considered to play key roles in the pathophysiology of depression [52, 53]. Additionally, inflammatory mediators (e.g., histamine) and cytokines (e.g., IFN-γ, IL-6) can trigger sleep disturbances because of their negative effects on the sleep–wake cycle and rapid eye-movement sleep [54, 55]. Sleep impairment may induce the development of depressed mood, resulting in suicidal ideation. Further studies are needed to explain the causal relationships between AR and RS-related cytokines, on the one hand, and mental health and HRQoL, on the other.

Before performing this study, we hypothesized that subjects with AR or RS might have poorer mental health and HRQoL than subjects with only AR or RS. Our hypothesis proved largely correct; we found that AR and RS exerted a negative synergistic impact on mental health (depressed mood and suicidal ideation) and HRQoL (pain/discomfort and anxiety/depression). Patients with both AR and RS exhibited more severe nasal and extra-nasal symptoms induced by both allergic reactions and RS-related inflammation. Additionally, allergic patients tend to be hypersensitive to somatic pain because pro-inflammatory cytokines induced by allergic processes play crucial roles in the modulation of central and peripheral nociception [56]. Thus, the subjective experience of somatic pain and discomfort in daily life may be exaggerated in such patients, as indicated by our findings.

In this study, AR and/or RS showed a negative impact on mental health and HRQoL. Because the patients with AR and/or RS may have several psychological problems, clinicians should thoroughly treat patients by easier and more comfortable methods. And they should also assess the patient’s psychological problems using validated tools. If the patients seems to have psychological problems, clinicians should help them receive psychological intervention.

A strength of our study is its design. As the KNHANES is a nationwide, complex, stratified, multistage probability-cluster survey of a representative sample of the non-institutionalized civilian population of Korea, the participants are representative the general Korean population. Second, the relatively large number of study participants (compared with other studies) afforded strong statistical power. Third, we considered a wide variety of data, including demographic characteristics, socioeconomic status, health behaviors, and other comorbidities, thus mitigating any probable bias by adjusting for confounding effects. Therefore, the association of AR and RS with mental health and HRQoL, as identified in this study, can be generalized. Last, we examined the mental health and HRQoL of subjects with both AR and RS; few previous studies have examined such subjects despite the fact that the diseases are frequently comorbid.

However, our study had several limitations. First, although this was a population-based nationwide study, a cross-sectional survey cannot identify causal relationships between AR and/or RS and mental health and HRQoL. Second, as diagnoses of AR and RS were self-reported, some recall bias may have been in play. However, other large population-based surveys used similar questionnaires to diagnose AR and sinus infections [57–59]. Third, the severity of both AR and RS may influence mental health and HRQoL, but we did not consider this possibility. Forth, the use of non-validated and simple questions limited our mental health survey methodologically. However, our single questionnaire was designed to cover the key criteria for major depressive episode in DSM-5 such as “2 weeks period” and “depressed mood” in A criteria and “functional impairment” in B criteria [60]. Our single question about suicidal ideation has been also known worldwide as one of the important tools to screen for the population vulnerable to suicide, even though the answer could have uncertainty about its reliability and validity.

## Conclusions

Our population-based, nationwide cross-sectional study showed that AR and RS synergistically compromised mental health and HRQoL. Importantly, combined AR and RS exhibited a strong association with suicidal ideation, which is the most dangerous consequence of poor mental health and also a major social problem. Therefore, physicians who manage patients with AR and/or RS should consider their mental health and HRQoL, especially when patients have both AR and RS.
